# Expression of miR-92a and miR-125b and Their Association with Chemoradiotherapy Response in Locally Advanced Cervical Cancer

**DOI:** 10.3390/ijms27041723

**Published:** 2026-02-11

**Authors:** Renny Anggia Julianti, Andi Darma Putra, Ahmad Fuady, Laila Nuranna, Gatot Purwoto, Kartiwa Hadi Nuryanto, Muhammad Yurizar Yudhistira

**Affiliations:** 1 Division of Gynecologic Oncology, Department of Obstetrics and Gynecology, Faculty of Medicine, Universitas Indonesia, Cipto Mangunkusumo Hospital, Central Jakarta 10430, Indonesia; andi.darma@ui.ac.id (A.D.P.); lailaril@gmail.com (L.N.); gatotpurwoto@gmail.com (G.P.); kartiwa_h_nuryanto@yahoo.com (K.H.N.); yuriyudhis@gmail.com (M.Y.Y.); 2 Department of Community Medicine, Faculty of Medicine, Universitas Indonesia, Central Jakarta 10310, Indonesia; ahmad.fuady01@ui.ac.id

**Keywords:** cervical cancer, chemoradiotherapy, miR-92a, miR-125b, treatment response

## Abstract

Cervical cancer remains a major cause of morbidity and mortality, with most cases in Indonesia diagnosed at a locally advanced stage. Although concurrent chemoradiotherapy is the standard treatment, response varies. Dysregulation of microRNAs (miRNAs), particularly oncogenic miR-92a and tumor suppressor miR-125b, may contribute to treatment resistance. This study aimed to evaluate the association between miR-92a and miR-125b expression and chemoradiotherapy response in locally advanced cervical cancer. This single-center retrospective cohort study included patients with stage IB3–IVA cervical cancer treated with chemoradiotherapy between 2019 and 2025. miRNA expression levels were measured from pretreatment tumor biopsy specimens. Poor response was defined as incomplete response or disease progression after treatment. Appropriate comparative, predictive, and survival analyses were performed. Sixty-eight patients were included. Poor response was significantly associated with underweight body mass index, elevated miR-92a, and reduced miR-125b expression (*p* < 0.05). High miR-92a and low miR-125b expression were also associated with shorter overall survival (*p* < 0.001). A combined model incorporating BMI, miR-92a, and miR-125b showed good predictive performance. Elevated miR-92a and reduced miR-125b are associated with poor treatment response and worse survival. These miRNAs may support risk stratification and treatment personalization in locally advanced cervical cancer.

## 1. Introduction

Cervical cancer remains the fourth most common cancer among women worldwide, with an estimated 661,021 new cases and 348,189 deaths in 2022 [1,2]. In Indonesia, cervical cancer is the second most frequently diagnosed malignancy, accounting for 36,964 cases and 20,708 deaths, reflecting a persistently high disease burden [3]. Most patients are diagnosed at a locally advanced stage; data from the FKUI–RSCM Cancer Registry show that 85.5% of cases present as locally advanced disease, with stage IIIC1, IIB, and IIIB being the most common. Concurrent chemoradiotherapy is the standard treatment for this population, yet despite reported complete response rates of approximately 70%, a substantial proportion of patients experience poor response, progression, or recurrence [4].

Resistance to chemoradiotherapy represents a major clinical challenge in locally advanced cervical cancer. Tumor response to radiation and chemotherapy depends largely on effective DNA damage induction and apoptotic signaling; however, tumor-specific molecular and epigenetic alterations may attenuate these processes and promote treatment resistance [5]. Beyond genetic changes, epigenetic regulation has emerged as a key contributor to therapeutic response through modulation of gene expression involved in cell cycle control, apoptosis, and DNA repair.

MicroRNAs (miRNAs) are small non-coding RNAs that act as key post-transcriptional regulators of cancer cell survival, invasion, and therapeutic response. Aberrant miRNA expression has been implicated in cervical carcinogenesis and treatment failure, particularly in patients receiving chemoradiotherapy [6,7,8,9]. A systematic review identified several miRNAs involved in cervical cancer progression, including miR-92a and miR-125b [9]. Beyond cervical cancer, expression of miR-92a and miR-125b has been associated with chemoresistance, recurrence, and metastasis in several solid malignancies, including pancreatic, hepatic, colorectal, non-small cell lung cancer, lung adenocarcinoma, and rectal adenocarcinoma [10,11,12,13,14,15,16,17,18,19,20,21,22,23,24].

However, despite their established biological relevance, the roles of miR-92 and miR-125b in modulating chemoradiotherapy response in cervical cancer remain insufficiently characterized. Moreover, the association between miR-92a and miR-125b expression levels and chemoradiotherapy response in locally advanced cervical cancer remains insufficiently explored, particularly in the Indonesian population. Therefore, this study aimed to evaluate differences in miR-92a and miR-125b expression according to chemoradiotherapy response in patients with locally advanced cervical cancer.

## 2. Results

This was a retrospective cohort study from 2019 to 2025. Archived samples from the biobank of patients diagnosed with cervical cancer since 2019 were included and followed until the study cut-off.

### 2.1. Baseline Demographic and Clinical Characteristics

Of 90 patients with locally advanced cervical cancer (stage IB3–IVA) receiving chemoradiotherapy at RSCM, 68 met the inclusion criteria. The mean age was 54.21 ± 10.22 years. Most patients were obese (35.3%) or underweight (25.0%). The majority were diagnosed at stage III (71.2%), followed by stage IV (20.6%). Squamous cell carcinoma was the predominant histopathological type (79.4%). The mean expression levels of miR-92a and miR-125b were 21.52 ± 2.02 and 20.90 ± 2.54, respectively, with a mean survival duration of 24.59 ± 17.44 months (Table 1).

### 2.2. Baseline Characteristics in Association with Treatment Response

Treatment response was not significantly associated with age, lymphovascular space invasion, tumor differentiation, or histopathology (*p* > 0.05). Significant associations were observed between treatment response and BMI, stage, miR-92a, miR-125b, and survival duration (all *p* < 0.05). Poor responders were more frequently underweight and had advanced-stage disease. They exhibited higher miR-92a expression, lower miR-125b expression, and shorter survival compared to good responders (*p* < 0.001) (Table 2).

### 2.3. Expression of miR-92a and miR-125b

**Figure 1:**
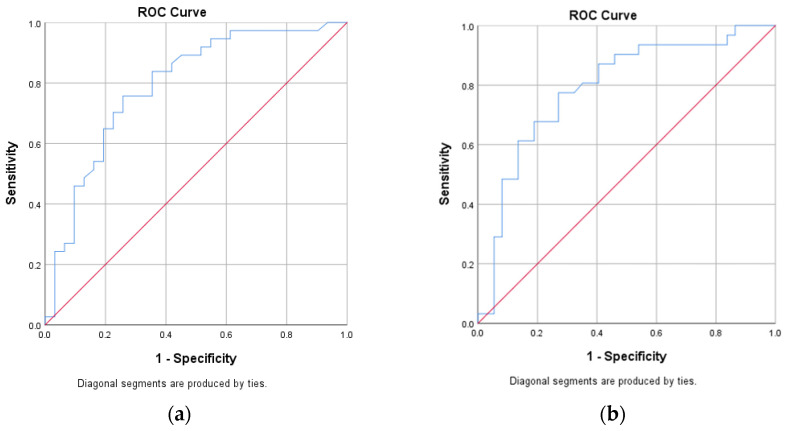
Receiver operating characteristic (ROC) curves of miR-92a for treatment response (**a**) and miR-125b for treatment response (**b**).

Receiver operating characteristic (ROC) analysis demonstrated that both miRNAs had moderate discriminatory ability for predicting treatment response. miR-92a yielded an AUC of 0.792 (95% CI: 0.682–0.902) with an optimal cut-off value of >21.32, providing a sensitivity of 75.7% and a specificity of 74.2%. miR-125b showed an AUC of 0.790 (95% CI: 0.680–0.901), with an optimal cut-off value of <20.78, yielding a sensitivity of 73.0% and a specificity of 77.4% (Figure 1, Table 3). These thresholds demonstrated balanced diagnostic performance and were significantly associated with poor treatment response on cross-tabulation analysis (*p* < 0.001).

### 2.4. Multivariate Analysis and Predictive Model

Variables with *p* < 0.05 in bivariate analysis were included in the multivariate logistic regression model. Underweight BMI (Adj OR = 13.63; *p* = 0.015), increased miR-92a expression (Adj OR = 2.16; *p* < 0.001), and miR-125b expression as a protective factor (Adj OR = 0.69; *p* = 0.009) were independently associated with treatment response (Table 4).

**Figure 2:**
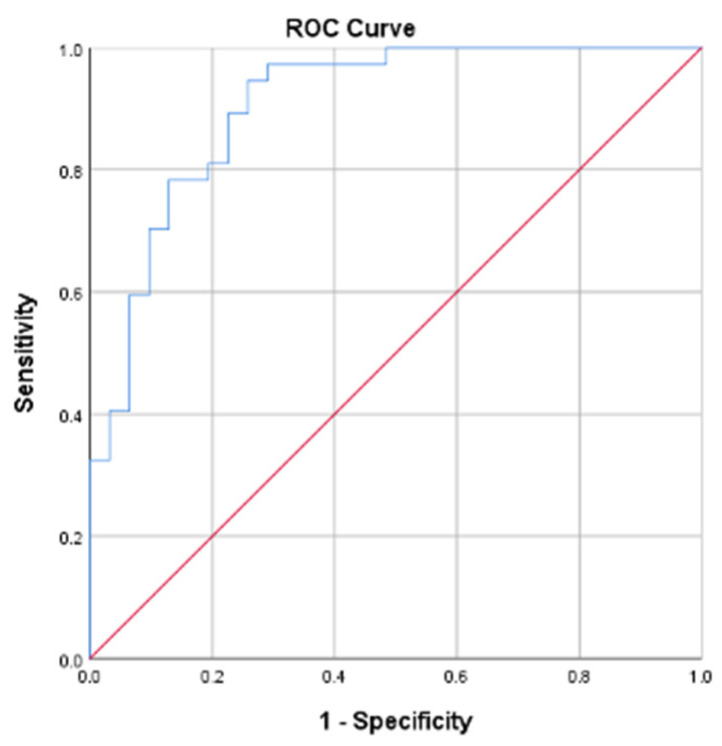
Receiver operating characteristic (ROC) curves of Probability Predictive Models.

The final predictive model demonstrated excellent discrimination, with an AUC of 0.906 (95% CI: 0.834–0.977). According to the Hosmer–Lemeshow criterion, a model is considered acceptable when the area under the curve exceeds 0.7. Overall classification accuracy was 79.4%, correctly identifying 77.4% of good responders and 81.1% of poor responders (Figure 2). Therefore, this model demonstrates excellent discriminative ability, indicating that treatment response can be reliably predicted using BMI, miR-92a, and miR-125b.

(1) $$\begin{array}{l} Treatment\;Response=Ln\left(\frac{P}{1-p}\right)\\ \;\;\;\;\;\;\;\;\;\;\;\;\;\;\;\;\;\;\;\;=-9.011+2.612\;BMI\;underweight\;\left(1\right)+0.680\;miR92a-0.369\;miR-125b<20.53\;(1) \end{array}$$

### 2.5. Comparative Analysis Between Expression of miR-92a, miR-125b and Baseline Characteristics

Higher miR-92a expression (≥21.32) was significantly associated with older age and more advanced FIGO stage (*p* < 0.05), but not with BMI, lymphovascular space invasion, tumor differentiation, or histopathological type. In contrast, lower miR-125b expression (<20.78) was significantly associated with underweight BMI (*p* = 0.004), while no significant associations were found with age, stage, lymphovascular space invasion, differentiation grade, or histopathology (Table 5).

### 2.6. Survival Analysis Between Expression of miR-92a and miR-125b

**Figure 3:**
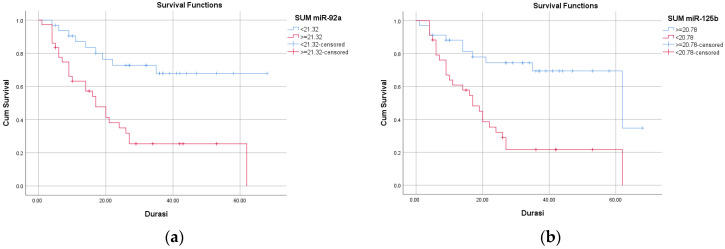
Kaplan–Meier curves of cumulative overall survival based on miR-92a expression (**a**), showing a hazard ratio (HR) of 3.67 (95% CI: 1.72–7.85; *p* = 0.001), and based on miR-125b expression (**b**), with an HR of 3.61 (95% CI: 1.74–7.51; *p* = 0.001).

Kaplan–Meier survival analysis revealed significantly reduced overall survival among patients with high miR-92a (≥21.32) and low miR-125b (<20.78) expression (log-rank *p* < 0.001). Patients with elevated miR-92a had a mean overall survival of 25.66 months compared with 51.41 months in those with lower expression (HR = 3.67; 95% CI: 1.72–7.85). Similarly, patients with low miR-125b expression had a mean overall survival of 24.35 months versus 49.69 months in those with higher expression (HR = 3.61; 95% CI: 1.74–7.51) (Figure 3, Table 6).

## 3. Discussion

The findings of this study suggest an association between miRNA expression and therapeutic outcome. Elevated miR-92a and reduced miR-125b expression identified patients at higher risk of poor treatment response and reduced overall survival, indicating their potential utility as minimally invasive predictive biomarkers.

### 3.1. Clinicopathological Factors and Treatment Response

Patients with poor response were predominantly underweight, had advanced FIGO stage (III–IV), and experienced shorter overall survival. Notably, underweight BMI remained an independent predictor of poor treatment response in multivariate analysis, indicating that nutritional and metabolic status exerts an effect on treatment efficacy beyond tumor stage alone.

From a biological perspective, underweight status reflects malnutrition, sarcopenia, and systemic inflammation, all of which are known to impair immune surveillance, DNA damage repair, and tolerance to cytotoxic therapy. Malnourished patients often exhibit reduced lymphocyte counts, impaired T-cell function, and altered cytokine profiles, which may compromise radiosensitivity and antitumor immune responses [25,26]. In addition, underweight patients are at higher risk of receiving suboptimal chemotherapy dosing due to concerns regarding toxicity, potentially leading to insufficient tumoricidal exposure [27]. These mechanisms provide a plausible explanation for the observed association between underweight BMI and poor chemoradiotherapy response, as reported in prior studies [28,29,30].

Conversely, excess adiposity has been linked to increased cervical cancer risk and may also influence treatment outcomes. Meta-analyses and large cohort studies have shown that overweight and obesity are associated with a modestly increased risk of cervical cancer, with risk estimates rising with increasing BMI [26,27,31]. Biologically, obesity is characterized by chronic low-grade inflammation, T-cell dysfunction, metabolic alterations, and dysregulated steroid hormone signaling, accompanied by increased levels of pro-inflammatory cytokines and adipokines implicated in tumor progression and epithelial–mesenchymal transition [32]. Moreover, concerns regarding chemotherapy toxicity may lead to dose reductions in patients with extreme body weight, potentially compromising treatment efficacy, underscoring current ASCO recommendations to use actual body weight for chemotherapy dosing in obese patients [25].

FIGO stage emerged as a critical determinant of treatment response, consistent with its established role as the strongest prognostic factor in cervical cancer [33]. Advanced-stage disease is characterized by larger tumor burden, hypoxic tumor microenvironments, increased genomic instability, and a higher likelihood of nodal and distant dissemination [34]. Tumor hypoxia reduces radiosensitivity by limiting oxygen-dependent DNA damage, while increased tumor heterogeneity and clonogenic cell populations contribute to treatment resistance. Large population-based and institutional studies have consistently demonstrated inferior local control, disease-free survival, and overall survival in patients with FIGO stage III–IV disease [35,36,37].

Precision medicine increasingly emphasizes population-specific molecular characteristics in cancer biology and treatment response. However, evidence regarding ethnic and racial differences in miRNA expression in gynecologic malignancies remains limited, as most available biomarker studies originate from European, American, and Asian populations [38,39]. Notably, data specifically addressing ethnic variation in miRNA expression within Asian populations with gynecologic malignancies are still lacking. To date, only a single large study in African American patients has reported race-specific differential expression of miRNAs, including miR-9 and miR-29, on breast invasive carcinoma, cervical squamous cell carcinoma, endocervical adenocarcinoma, ovarian serous cystadenocarcinoma, uterine corpus endometrial carcinoma, and uterine carcinosarcoma [40].

### 3.2. Expression of miR-92a and miR-125b as Predictors of Treatment Response

This study identified higher miR-92a expression and lower miR-125b expression in patients with poor response. Diagnostic analyses showed that both miRNAs exhibited moderate-to-good discriminative performance, supporting their potential utility as predictive biomarkers of chemoradiotherapy response.

The oncogenic role of miR-92a in cervical cancer is well documented. Elevated miR-92a expression has been associated with tumor progression, lymph node metastasis, poor differentiation, and resistance to cisplatin-based therapy, primarily through suppression of key tumor-suppressor pathways involved in cell cycle regulation and survival, such as FBXW7 and PI3K-related signaling [41,42,43,44,45,46,47,48,49]. The present findings are consistent with these mechanistic studies and further support the clinical relevance of miR-92a as a predictor of therapeutic resistance.

Conversely, miR-125b is recognized as a tumor-suppressive miRNA. Reduced miR-125b expression has been linked to cervical carcinogenesis and angiogenesis, largely through PI3K/AKT pathway activation mediated by VEGF upregulation [50,51,52,53]. Experimental evidence shows that miR-125b overexpression inhibits tumor cell proliferation and promotes apoptosis in cervical cancer models, reinforcing its protective role [52]. The observed downregulation of miR-125b in poor responders in this study aligns with these data and suggests its involvement in treatment sensitivity.

### 3.3. Association of miRNA Expression with Patient Characteristics

In this study, miR-92a expression was significantly associated with patient age, with higher expression observed in older patients. This finding is biologically plausible, as miR-92a is part of the miR-17–92 cluster, which regulates cell cycle progression, senescence, and apoptosis through interactions with E2F transcription factors and the p53 pathway. Age-related alterations in miRNA expression profiles, including increased miR-92a levels, have been reported across multiple tissues, although the direction and magnitude of change appear to be tissue-specific [54].

Additionally, miR-125b expression was significantly associated with BMI, with lower levels observed in underweight patients. The miR-125 family plays a key role in adipogenesis, energy metabolism, inflammatory signaling, and mitochondrial function through pathways involving STAT3, PI3K/AKT, FOXO1, and PPAR signaling. These findings suggest that systemic metabolic status may influence miRNA expression and, consequently, tumor behavior and treatment response [32,55].

### 3.4. Strengths, Limitations, and Remaining Gaps

This study has several notable strengths. It focuses on miR-92a and miR-125b, two molecular biomarkers that remain relatively underexplored in locally advanced cervical cancer, particularly in the context of chemoradiotherapy response. The integration of molecular data with clinicopathological variables provides a more comprehensive framework for understanding treatment resistance. Additionally, the use of standardized treatment protocols, objective response assessment based on RECIST criteria, and the incorporation of survival analysis enhance the clinical relevance and robustness of the findings.

Several limitations should be acknowledged. The observational design precludes causal inference between miRNA expression and treatment response, and the relatively small sample size may limit statistical power and generalizability. The study population was restricted to Indonesian patients, representing an Asian population, which may limit applicability to other ethnic groups. Technical and biological variability in miRNA measurement may introduce bias despite standardized procedures. In addition, the analysis focused on only two miRNAs and did not capture the broader regulatory network involved in chemoradiotherapy resistance. The short follow-up period also limited the assessment of long-term outcomes such as recurrence.

Future studies should include external validation in larger, multi-center cohorts with diverse populations, longer follow-up, and broader miRNA profiling to better elucidate the role of miRNAs in chemoradiotherapy resistance.

### 3.5. Clinical Implication

In clinical practice, miRNA-based risk stratification could support personalized treatment planning, closer surveillance, nutritional optimization, and consideration of alternative or intensified therapeutic strategies in high-risk patients prior to treatment initiation.

## 4. Materials and Methods

### 4.1. Study Design

This study employed a single-center retrospective cohort design to evaluate chemoradiotherapy response as the primary outcome. Patient recruitment was conducted retrospectively by identifying eligible cervical cancer cases treated with definitive concurrent chemoradiotherapy at Dr. Cipto Mangunkusumo National Referral Hospital (RSCM), Jakarta, Indonesia, between 2019 and 2025. The study was conducted following approval from the institutional ethics committee of the Faculty of Medicine, Universitas Indonesia–RSCM (KET-740/UN2.F1/ETIK/PPM.00.02/202).

Pretreatment cervical tumor biopsy specimens were obtained at the time of initial diagnosis, prior to the initiation of chemoradiotherapy, and were stored for subsequent molecular analysis. Laboratory analysis of miR-92a and miR-125b expression was performed after case identification, following completion of clinical data collection. Clinical, pathological, treatment, and outcome data were retrieved from medical records and institutional databases. Treatment response was assessed as the primary outcome at a standardized time point of 4–12 weeks after completion of chemoradiotherapy, in accordance with institutional follow-up protocols, to ensure consistency across patients.

### 4.2. Study Population

The target population included all women diagnosed with cervical cancer (ICD-10 C53) at RSCM, while the accessible population consisted of patients with locally advanced cervical cancer stages IB3, IIA2, and IIB–IVA who underwent definitive chemoradiotherapy. Eligible participants were women with histopathologically confirmed squamous cell carcinoma, adenocarcinoma, or adenosquamous carcinoma who completed standard chemoradiotherapy and had available cervical biopsy specimens preserved as fresh-frozen tissue or formalin-fixed paraffin-embedded blocks suitable for RNA extraction. Patients with distant metastases, active infections, metabolic disorders, severe systemic comorbidities, prior radical surgery, recurrent disease, synchronous malignancies, use of other chemotherapy or immunosuppressive agents, or incomplete treatment were excluded.

### 4.3. Treatment Protocols

All patients received standardized external beam radiotherapy with a total dose of 50 Gy delivered in 1.8 Gy fractions, five days per week for five weeks, concurrently with weekly cisplatin at 40 mg/m^2^, contingent upon adequate renal function. This was followed by intracavitary brachytherapy administered over three to four weeks at 7–8 Gy per fraction. Radiation fields included the primary tumor and regional lymph nodes, and uniform protocols were applied to minimize therapeutic variability. Treatment response was assessed 4–12 weeks after therapy completion using RECIST version 1.1 criteria based on clinical examination, ultrasonography, and pelvic MRI. Responses were classified as good (complete or partial response) or poor (stable or progressive disease). To complement short-term response evaluation, overall survival analysis was incorporated.

### 4.4. Molecular Analysis

Expression levels of miR-92a and miR-125b were quantified using real-time reverse transcription polymerase chain reaction (RT-qPCR). Total RNA was extracted from fresh-frozen cervical biopsy specimens using the miRNeasy Kit (Qiagen, Germantown, MD, USA) distributed from GeneCraft Labs in Indonesia. RNA concentration and purity were assessed by NanoDrop spectrophotometry. Reverse transcription and amplification were performed using TaqMan^®^ MicroRNA assays on an Applied Biosystems real-time PCR platform. All samples were analyzed in duplicate, with U6 small nuclear RNA used as an endogenous control for normalization. Relative expression levels were calculated using the ΔCt and ΔΔCt methods, with fold changes determined by the 2^−ΔΔCt^ formula. Optimal cut-off values were established through receiver operating characteristic (ROC) curve analysis.

### 4.5. Statistical Analysis

Clinical and molecular data were recorded using standardized case report forms. Descriptive statistics are presented as means with standard deviations, medians with interquartile ranges, or frequencies, as appropriate. Data normality was assessed prior to analysis. Bivariate analyses were performed using independent *t*-tests or Mann–Whitney U tests for numerical variables and chi-square or Fisher’s exact tests for categorical variables. Multivariate binary logistic regression was conducted to identify independent predictors of poor treatment response. Survival analysis was performed using Kaplan–Meier curves with log-rank tests, and Cox proportional hazards regression was used to estimate hazard ratios with 95% confidence intervals. Statistical significance was defined as *p* < 0.05.

## 5. Conclusions

Factors such as underweight BMI, advanced FIGO stage, elevated miR-92a, and reduced miR-125b expression were independently associated with poor chemoradiotherapy response and inferior survival outcomes. These results support the role of miR-92a and miR-125b as biologically relevant biomarkers in chemoradioresistance. Incorporating molecular biomarkers into conventional staging systems may enhance prognostic accuracy and guide individualized management strategies for patients with locally advanced cervical cancer.

## Figures and Tables

**Table 1:** Study population and baseline characteristics.

Variables	Total	
	n	%
Age (Mean ± SD)	54.21	±10.22
Body mass index		
Underweight (<18.5 kg/m^2^)	17	25.0%
Normoweight (18.5–<23.0 kg/m^2^)	15	22.1%
Overweight (>23.0–<25.0 kg/m^2^)	12	17.6%
Obese (>25 kg/m^2^)	24	35.3%
FIGO 2018 Stage		
I and II	5	7.4%
III	49	72.1%
IV	14	20.6%
Lymphovascular Space Invasion (LVSI)		
No	66	97.1%
Yes	2	2.9%
Tumor differentiation		
Poorly differentiated	11	16.2%
Moderately differentiated	33	48.5%
Well-differentiated	24	35.3%
Histopathology		
Squamous cell carcinoma	54	79.4%
Adenocarcinoma	14	20.6%
miR-92a (Mean ± SD)	21.52	±2.02
miR-125b (Mean ± SD)	20.90	±2.54
Survival (months) (Mean ± SD)	24.59	±17.44

**Table 2:** Comparative analysis between baseline characteristics and treatment response.

Variables	Treatment Response				*p*-Value
	Good n = 31		Poor n = 37		
	n	%	n	%	
Age (Mean ± SD) ^a^	51.58	±9.03	56.41	±10.75	0.052
Body mass index ^b^					<0.001 *
Underweight (<18.5 kg/m^2^)	2	6.5%	15	40.5%	
Normoweight (18.5–<23.0 kg/m^2^)	5	16.1%	10	27.0%	
Overweight (>23.0–<25.0 kg/m^2^)	8	25.8%	4	10.8%	
Obese (>25 kg/m^2^)	16	51.6%	8	21.6%	
FIGO 2018 Stage ^c^					0.016 *
I and II	5	16.1%	0	0.0%	
III	24	77.4%	25	67.6%	
IV	2	6.5%	12	32.4%	
Lymphovascular Space Invasion (LVSI) ^c^					0.496
No	31	100.0%	35	94.6%	
Yes	0	0.0%	2	5.4%	
Tumor differentiation ^b^					0.093
Poorly differentiated	2	6.5%	9	24.3%	
Moderately differentiated	16	51.6%	17	45.9%	
Well-differentiated	13	41.9%	11	29.7%	
Histopathology ^b^					0.710
Squamous cell carcinoma	24	77.4%	30	81.1%	
Adenocarcinoma	7	22.6%	7	18.9%	
miR-92a (Mean ± SD) ^b^	20.49	±1.84	22.38	±1.75	<0.001 *
miR-125b (Mean ± SD) ^b^	22.04	±2.13	19.95	±2.49	<0.001 *
Survival (months) (Mean ± SD) ^b^	33.16	±15.63	17.41	±15.69	<0.001 *

Notes: ^a^ independent *t*-test (normally distributed numerical data); ^b^ Mann–Whitney U test (ordinal data and non-normally distributed numerical data); ^c^ chi-square or Fisher’s exact test (nominal data); * statistically significant at *p* < 0.05.

**Table 3:** Expression of miR-92a and miR-125b.

Variables	Area Under Curve	95% Confidence Interval	Cut-Off	Sensitivity	Specificity	Positive Predictive Value	Negative Predictive Value
miR-92a	0.792	0.682–0.902	>21.32	75.7%	74.2%	77.8%	71.9%
miR-125b	0.790	0.680–0.901	<20.78	73.0%	77.4%	79.4%	70.6%

**Table 4:** Results of multivariable regression and prediction model.

Variables	Crude OR	95%CI		*p*-Value	Adj OR	95%CI		*p*-Value
		Lower	Upper			Lower	Upper	
Age	1.05	1.00	1.11	0.058	n/s			
Body mass index (Underweight)	9.89	2.04	47.81	0.004 *	13.63	1.67	111.43	0.015 *
Tumor differentiation (Poorly differentiated)	4.66	0.92	23.50	0.062	n/s			
Histopathology (Adenocarcinoma)	0.80	0.25	2.60	0.710	n/s			
miR-92a	1.89	1.32	2.69	<0.001 *	2.16	1.42	3.27	<0.001 *
miR-125b	0.66	0.51	0.86	0.002 *	0.69	0.52	0.91	0.009 *

Note: Logistic regression; OR = Odd Ratio; CI = Confidence interval; * significant *p* < 0.05.

**Table 5:** Comparison between expression of miR-92a, miR-125b and baseline characteristics.

Variables	miR-92a				*p*-Value	miR-125b				*p*-Value
	<21.32 n = 32		≥21.32 n = 36			≥20.78 n = 34		<20.78 n = 34		
	n	%	n	%		n	%	n	%	
Age (mean ± sd) ^a^	51.28	±10.93	56.81	±8.91	0.025 *	53.12	10.19	55.29	10.29	0.384
BMI ^b^					0.583					0.004 *
Underweight	7	21.9%	10	27.8%		4	11.8%	13	38.2%	
Normoweight	7	21.9%	8	22.2%		6	17.6%	9	26.5%	
Overweight	6	18.8%	6	16.7%		8	23.5%	4	11.8%	
Obese	12	37.5%	12	33.3%		16	47.1%	8	23.5%	
FIGO stage ^b^					0.015 *					0.242
I	0	0.0%	1	2.8%		0	0.0%	1	2.9%	
II	4	12.5%	0	0.0%		3	8.8%	1	2.9%	
III	25	78.1%	24	66.7%		26	76.5%	23	67.6%	
IV	3	9.4%	11	30.6%		5	14.7%	9	26.5%	
LVSI ^c^					0.494					1.000
No	32	100.0%	34	94.4%		33	97.1%	33	97.1%	
Yes	0	0.0%	2	5.6%		1	2.9%	1	2.9%	
Differentiation ^b^					0.381					0.263
Poorly differentiated	3	9.4%	8	22.2%		3	8.8%	8	23.5%	
Moderately differentiated	17	53.1%	16	44.4%		18	52.9%	15	44.1%	
Well-differentiated	12	37.5%	12	33.3%		13	38.2%	11	32.4%	
Histopathology					0.724					1.000
Squamous cell carcinoma	26	81.3%	28	77.8%		27	79.4%	27	79.4%	
Adenocarcinoma	6	18.8%	8	22.2%		7	20.6%	7	20.6%	

Notes: ^a^ independent *t*-test (normally distributed numerical data); ^b^ Mann–Whitney U test (ordinal data); ^c^ chi-square or Fisher’s exact test (nominal data); * statistically significant at *p* < 0.05.

**Table 6:** Comparison of Survival Based on miR-92a and miR-125b Expression Levels.

Expression miRNA	Total N	N of Events	Censored		Mean	95% CI		Log Rank
			N	Percent		Lower	Upper	*p*-Value
miR-92a < 21.32	32	9	23	71.9%	51.41	42.31	60.50	<0.001 *
miR-92a ≥ 21.32	36	28	8	22.2%	25.66	17.92	33.39	
Overall	68	37	31	45.6%	36.47	30.05	42.89	
miR-125b ≥ 20.78	34	10	24	70.6%	49.69	40.71	58.68	<0.001 *
miR-125b < 20.78	34	27	7	20.6%	24.35	16.88	31.82	
Overall	68	37	31	45.6%	36.47	30.05	42.89	

Note: Log Rank; CI = Confidence interval; * statistically significant at *p* < 0.05.

## Data Availability

The original contributions presented in this study are included in the article. Further inquiries can be directed to the corresponding author.
